# Genetic Analysis of the Coronavirus SARS-CoV-2 Host Protease *TMPRSS2* in Different Populations

**DOI:** 10.3389/fgene.2020.00872

**Published:** 2020-08-04

**Authors:** Roberta Russo, Immacolata Andolfo, Vito Alessandro Lasorsa, Achille Iolascon, Mario Capasso

**Affiliations:** ^1^Dipartimento di Medicina Molecolare e Biotecnologie Mediche, Università degli Studi di Naples Federico II, Naples, Italy; ^2^CEINGE Biotecnologie Avanzate, Naples, Italy

**Keywords:** *TMPRSS2*, COVID-19, SARS-CoV-2, genetic population analysis, eQTL, variant

## Introduction

In December 2019, a new infectious respiratory disease emerged in Wuhan, Hubei province, China (Huang et al., 2020; Wang et al., 2020; Zhu et al., 2020). It diffused rapidly worldwide and became a pandemic. The World Health Organization (WHO) has officially named it coronavirus disease 2019 (COVID-19), and the virus has been classified as severe acute respiratory syndrome coronavirus 2 (SARS-CoV-2). COVID-19 causes a severe clinical picture, ranging from mild malaise to death by sepsis/acute respiratory distress syndrome (Gabutti et al., 2020). The epidemiology of COVID-19 highlights differences either in the susceptibility to the infection or in death rates among populations (https://covid19.who.int/). As for other multifactorial conditions, this variability may be related to environmental differences among countries, such as access to medical care and the age structure of the population (Pareek et al., 2020); nevertheless, it may be related also to human genetic variability. From this perspective, *angiotensin-converting enzyme 2* (*ACE2)* and *serine protease 2* (*TMPRSS2)* genes are good candidates due to their role in the viral infection. Indeed, ACE2 was reported to be the main entry receptor for SARS-CoV-2 (Wang et al., 2020). Entry depends on the binding of the surface unit, S1, of the spike (S) protein of the virus to the receptor. SARS-CoV-2 engages ACE2 as the entry receptor and employs the host cellular TMPRSS2 for S-protein priming (Matsuyama et al., 2010; Hoffmann et al., 2020). TMPRSS2 is important for the spread of several viruses, including influenza A viruses and other coronaviruses (Glowacka et al., 2011; Gierer et al., 2013; Zhou et al., 2015; Shirato et al., 2016, 2018; Iwata-Yoshikawa et al., 2019).

Different studies have already investigated the potential associations between genetic variants of the *ACE2* gene and COVID-19 (Asselta et al., 2020; Benetti et al., 2020; Cao et al., 2020; Darbani, 2020; Devaux et al., 2020). Here, we have analyzed the genetic markers of the *TMPRSS2* gene and the differences in their alternative allele frequencies (AFs) among populations to identify possible susceptibility loci to COVID-19 and to correlate them with disease epidemiology.

## Tissue Expression of *TMPRSS2* Gene

*TMPRSS2* is highly expressed in ileal absorptive enterocytes, nasal goblet secretory cells, epithelial cells of bronchi, as well as type I and type II alveolar cells (Bertram et al., 2012; Collin et al., 2020; Ziegler et al., 2020). *TMPRSS2* expression in type I alveolar cells increases with aging in mice and humans (Schuler et al., 2020). Additionally, *TMPRSS2* is highly expressed in corneal epithelium and conjunctival specimens, suggesting that ocular surface cells could be the gateway of SARS-CoV-2 as well as a reservoir for person-to-person transmission (Collin et al., 2020).

Accordingly, *in-silico* expression analysis of the *TMPRSS2* supported the high *TMPRSS2* expression in tissues of the respiratory tract, such as the bronchus, pharyngeal mucosa, and lung. However, no difference in gene expression between males and females was observed for non-gender-specific tissues (Figures S1A,B).

## Analysis of Genetic Variants of *TMPRSS2* Locus

We analyzed 1,025 variants in the *TMPRSS2* gene region (chr21:42836478-42903043, 66.566 Kb) from the gnomAD v2.1.1 database. Annotation of *TMPRSS2* variants was performed with ANNOVAR by using 34 pathogenic variant scores (Table S1) and the AFs of 17 populations (Table S5; Russo et al., 2020). Genomic coordinates were based on the GRCh37/hg19 build. The classification of non-synonymous variants was performed using the following predictor tools: M-CAP (score >0.025), MutationTester (A-D, disease-causing), CADD v1.3 (Phred score >15) for the pathogenic variants. VEST3 (score <0.5), REVEL (score <0.5), and RadialSVM (T, tolerated) for the benign variants (Rajarshi et al., 2017). Variants with conflicting interpretations were excluded from further analyses.

**Figure 1:**
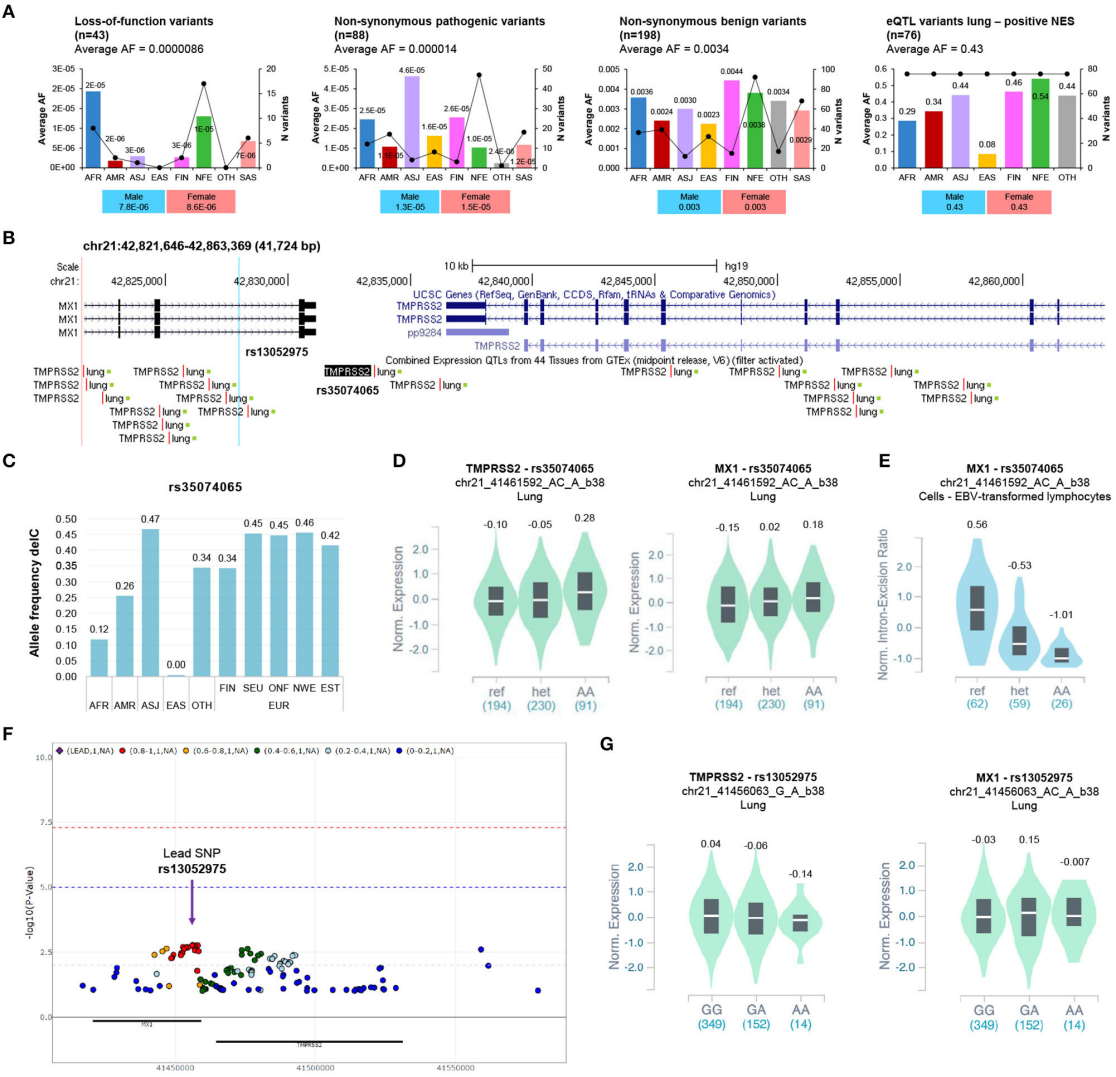
Analysis of the coding variants and the eQTLs of *TMPRSS2* locus. **(A)** The alternative allele frequency (AF) distribution of non-synonymous pathogenic, benign, loss-of-function, and eQTL-lung variants (positive NES) of *TMPRSS2* in different populations. The Y axis of each bar plot shows average allele frequency (AF) in each population. The second right axis displays the number of variants within each population. The bar graph below shows the AF distribution in the overall gnomAD population stratified according to gender. AFR, African/African American; AMR, Latino/Admixed American; ASJ, Ashkenazi Jewish; EAS, East Asian; FIN, Finnish; NFE, Non-Finnish European; SAS, South Asian; OTH, Other (population not assigned). **(B)** Schematics of the genomic region encompassing eQTL lung variants of *TMPRSS2* locus (NES positive ≥ 0.1) by Genome Browser (GRCh37/hg19, https://genome.ucsc.edu/). The most significant eQTL-lung (rs35074065) and the eQTL rs13052975, associated with severe COVID-19, are highlighted. **(C)** The allele frequencies of delC variant rs35074065 were annotated by the gnomAD database (WGS data). AFR, African/African American; AMR, Latino/Admixed American; ASJ, Ashkenazi Jewish; EAS, East Asian; OTH, Other (population not assigned); FIN, Finnish; SEU, Southern European; ONF, Other non-Finnish European; NEW, North-Western European; EST, Estonian. **(D)** Violin plot showing the effect of the eQTL rs35074065 on *TMPRSS2* and *MX1* expression in lung (*TMPRSS2*: *p* = 3.9e-11; NES = 0.13; *MX1*: *p* = 0.000010; NES = 0.20). **(E)** Violin plot showing the effect of the sQTL rs35074065 on *MX1* splicing isoform expression (*p* = 1.6e-13; NES = −0.83). **(F)** Regional association plot (*P* < 0.1) of variants at *TMPRSS2* locus (proximity: ± 50Kb; analysis II, adjusted for top 10 PCs, age, sex) and respiratory failure in COVID-19 (data from COVID-19 GWAS results browser, https://ikmb.shinyapps.io/COVID-19_GWAS_Browser/). **(G)** Violin plot showing the effect of the eQTL rs13052975 on *TMPRSS2* and *MX1* expression in lung (*TMPRSS2*: *P* = 0.0063; NES = −0.072; *MX1*: *P* = 0.15; NES = −0.090).

The locus region comprises 496 non-coding and 520 coding variants (Table S2). Forty-three variants were classified as loss-of-function (LoF), while 88/334 (26%) non-synonymous variants were predicted as pathogenic. All of them exhibit very low AFs (Figure 1A). Accordingly, a recent study identified a few functional ultra-rare variants in *TMPRSS2*, all of them with AFs < 0.001 (Gupta et al., 2020). These findings agree with the recommended benign frequency cut-off of 0.0001 for the *TMPRSS2* gene, as from the Varsome database.

Africans (AFR) showed the highest AFs for the LoF variants across populations. Similarly, the Swedish population exhibited the highest AF for LoF variants among the Europeans (Figure S2A). Regarding the non-synonymous pathogenic variants, we observed the highest AF among the Ashkenazi Jewish (ASJ) population (Figure 1A), while the Finnish (FIN) showed the highest AF among European subpopulations (Figure S2A). The AFs of non-synonymous variants classified as benign (198/334, 59.3%) were similarly distributed among the different populations (Figure 1A).

## Analysis of the Genetic Regulatory Variants Driving *TMPRSS2* Expression

To analyze the distribution of expression quantitative trait loci (eQTL) for *TMPRSS2*, we used the data from the Genotype Tissue Expression (GTEx) database (Table S3). The reference transcript for *TMPRSS2* annotation was NM_001135099 (ENST00000398585) (Figure S1C).

We found 203 unique and significant (FDR<0.05) eQTLs for *TMPRSS2* in five different tissues: 136 (66.9%) in lung, 56 (27.6%) in testis, 9 (4.4%) in prostate, 1 (0.5%) in ovarian, and 1 in thyroid (0.5%) tissue. The AFs of the 136 eQTLs with the strongest association in the lung tissue (eQTLs-lung) showed no substantial differences among different populations (Figure S2B). Nevertheless, the average AF of 76 eQTLs-lung with a positive normalized effect size (NES) was higher in European populations (FIN, 0.463; NFE, 0.541) compared to the average AF observed in the East Asian population (EAS) (0.085) (Figure 1A, Table S4). Of note, Europe displayed the highest median prevalence of COVID-19 cases among the WHO regions, while South East Asia showed a low prevalence of the disease (Figure S3).

Interestingly, the top 25 variants (NES > 0.1) localize in a genomic region that includes both *TMPRSS2* and *MX1* genes. The most significant eQTL, rs35074065, is in the intergenic region between the two genes (distance = 2379 from *MX1*; distance = 2958 from *TMPRSS2*) (Figure 1B) and shows the lowest AF in EAS (delC, 0.0049) (Figure 1C). Notably, the alternative allele delC seems to be associated with high expression of both *TMPRSS2* and *MX1* in lung tissue (Figure 1D). Moreover, the same variant is also a splicing QTL (sQTL) associated with low expression of *MX1* splicing isoform in different tissues (Figure 1E).

A recent analysis of nasal gene expression and genome-wide genetic variation data yielded three independent *TMPRSS2* eQTLs located in the downstream region of the gene: the SNP rs1475908, whose alternative allele (A) is associated with low *TMPRSS2* expression, and the two variants rs74659079 (allele T) and rs2838057 (allele A), both associated with high *TMPRSS2* expression. Interestingly, the eQTL rs1475908 shows the highest AF among EAS (A:0.38) and EUR (A:0.35) and the lowest frequency among Latinos (0.17) (Sajuthi et al., 2020).

These findings agree with a previous study that demonstrated the association of two high *TMPRSS2* expression-variants, rs2070788 (allele G) and rs383510 (allele T), with increased susceptibility to the influenza virus infection A (H7N9) (Cheng et al., 2015). Of note, the SNP rs2070788 was recently included in a haplotype associated with high *TMPRSS2* expression, whose AF is significantly increased in Europeans (Asselta et al., 2020).

We also verified the association between variants at *TMPRSS2* locus and respiratory failure in patients with COVID-19 using the summary statistics of a recently published GWAS (Ellinghaus et al., 2020). We found 13 SNPs showing a level of significance less than or equal to 0.002 in high-linkage disequilibrium (LD) (r2:0.8-1) and a total 89 SNPs with *P* < 0.05 (Figure 1F, Table S6).

The most significant eQTL-lung for TMPRSS2 (rs35074065) was found to be not significant (*P* = 0.30, OR = 1.07) and was located outside the LD block of the most significant rs13052975 variant (Figure S4, Table S6), whose alternative allele (A) correlates with low *TMPRSS2* in the lung (*p* = 0.0063), albeit with a non-significant *p*-value after multiple testing correction (Figure 1G). However, while current GWAS results do not support a role for the eQTL in respiratory failure associated with COVID-19, we cannot exclude its role in general susceptibility or other definitions of severity, such type of symptoms shown and duration of illness.

## Discussion

TMPRSS2 plays an important role in initiating SARS-CoV-2 and other respiratory viral infections (Glowacka et al., 2011). It has been suggested recently that the SARS-CoV-2 sequence has evolved by generating a unique four amino acid insertion between S1 and S2 domains of the spike protein, which created potential furin or TMPRSS2 cleavage site (Wu et al., 2020).

*TMPRSS2* is highly expressed in tissues of the aerodigestive tract (Bertram et al., 2012; Collin et al., 2020; Ziegler et al., 2020). Epidemiological data showed that the incidence and severity of diagnosed COVID-19 may be higher in men than women. Nevertheless, *TMPRSS2* gene expression data from the GTEx database do not highlight any difference between males and females. Importantly, developmental regulation of the expression of the *TMPRSS2* gene has been suggested that may underlie the relative protection of infants and children from severe respiratory illness (Schuler et al., 2020).

To investigate the genetic features of *TMPRSS2* locus among different populations, we analyzed both the coding-region variants of *TMPRSS2* and the eQTLs, which may regulate its gene expression. Our findings do not support the existence of common coding pathogenic variants for TMPRSS2 among different populations. Accordingly, recent studies identified only a few pathogenic, ultra-rare variants in this gene (Gupta et al., 2020; Paniri et al., 2020; Sajuthi et al., 2020). Therefore, given their rarity, we do not believe that coding variants, except for some rare cases, can determine the diverse susceptibility to viral infection and diverse clinical manifestations.

Conversely, it seems that the genetic regulatory variants driving *TMPRSS2* expression may have a role in the different susceptibility to SARS-CoV-2 and other respiratory viral infections among populations. Our *in-silico* analysis shows that common eQTLs-lung for *TMPRSS2* are less frequent in EAS but more frequent in EUR. Interestingly, the allele delC of the top significant eQTL (rs35074065) associates with higher expression of *TMPRSS2* compared to the reference allele and is less frequent among East Asians, suggesting a protective role against the infection in this population. The potential protective role of genetic variants associated with reduced *TMPRSS2* expression is supported by a GWAS. This demonstrated that rs35074065 is near to another eQTL, rs13052975, nominally significantly associated with severe COVID-19 (Ellinghaus et al., 2020). The alternative allele (A) of rs13052975 confers protection against severe clinical phenotype of the disease, correlates with low expression of *TMPRSS2*, and is common among EAS (A:0.45), while its frequency in other populations is below 0.26 (AFR, A:0.26; AMR, A:0.21; NFE, A:0.16).

Interestingly, the top eQTL variants derived from the GTEx database were in the intergenic region between *TMPRSS2* and *MX1* genes. This locus has already been shown to harbor common genetic variants with pleiotropic effects on age-related diseases like heart failure, stroke, coronary heart disease, and atrial fibrillation (He et al., 2016). *MX1* is an interferon (IFN)-α/β-inducible gene that codifies a guanosine triphosphate-metabolizing protein involved in the cellular antiviral response. It is widely recognized as an influenza susceptibility gene (Ciancanelli et al., 2016). Of note, the downregulation of *MX1* has been documented in non-responder patients to interferon-based antiviral therapy of chronic hepatitis C virus infection (Persico et al., 2008). GTEx data suggested that the eQTL rs35074065 (delC) is associated with high expression of *TMPRSS2* but also with a low expression of the *MX1*-splicing isoform (ENSG00000157601.13) in the esophagus, LCL, adipose tissue, whole blood, breast, small intestine, and lung (P ranging from 2 × 10–15 to 6 × 10–7). However, the strongest *MX1*-splicing QTLs at these tissues (P ranging from 2 × 10–45 to 1.9 × 10–16) were located within the coding region of *MX1*, and independent analyses thus need to be performed to verify if the effect of intergenic variant rs35074065 on *MX1* isoform still remains significant after accounting for the stronger variants.

We hypothesize that common variants driving *TMPRSS2* expression might have a mild-to-moderate effect in the susceptibility to SARS-CoV-2 infection. Particularly, genetic variants associated with reduced *TMPRSS2* expression might confer less individual susceptibility to SARS-CoV-2 infection and favor a better outcome. This hypothesis agrees with epidemiological data that show higher COVID-19 prevalence and mortality rates in Europe and the Americas (with a greater frequency of high-*TMPRSS2* expression-associated alleles). Conversely, a lower prevalence of the disease and mortality rates are found in South East Asia characterized by a high frequency of low-*TMPRSS2* expression-associated alleles. However, genetic studies in large cohorts of COVID-19 cases and appropriate controls are needed to confirm this hypothesis.

Unraveling the role of regulatory variants of this locus could represent an interesting starting point for the treatment of COVID-19. Indeed, targeting TMPRSS2 expression and/or activity could be a promising candidate for potential interventions against COVID-19 (Kawase et al., 2012; Stopsack et al., 2020). Of note, the camostat mesylate (CM), a serine protease inhibitor that blocks TMPRSS2 activity, has been already introduced in Japan for the treatment of unrelated disorders (Gierer et al., 2013; Zhou et al., 2015). CM showed a protecting role against death in mice following a lethal SARS-CoV infection. Moreover, the advantage is its low cost (Uno, 2020). Unfortunately, to date, there are no clinical data on CM in COVID-19, and so human clinical trials are desirable.

## Author Contributions

IA, RR, and MC designed and conducted the study, and prepared the manuscript. MC, VL, and RR analyzed the data. AI provided a critical review of the manuscript. All the authors read and approved the final manuscript.

## Conflict of Interest

The authors declare that the research was conducted in the absence of any commercial or financial relationships that could be construed as a potential conflict of interest.
