# Supplementary material for: Genetic Analysis of the Coronavirus SARS-CoV-2 Host Protease TMPRSS2 in Different Populations
Source: Front Genet. 2020 Aug 4;11:872. doi: 10.3389/fgene.2020.00872 (PMC7417663; doi:10.3389/fgene.2020.00872)
Supplement: Supplementary file 7 [file Data_Sheet_1.pdf]

SUPPLEMENTAL DATA

Article title:

Genetic analysis of the novel coronavirus SARS-CoV-2 host protease *TMPRSS2* in different populations

Authors:

Roberta Russo, Immacolata Andolfo, Alessandro Vito Lasorsa, Achille Iolascon, Mario Capasso

Corresponding author:

Mario Capasso, Dipartimento di Medicina Molecolare e Biotecnologie Mediche, Università degli Studi di Napoli Federico II, Napoli, Italy; CEINGE Biotecnologie Avanzate, Napoli, Italy; mario.capasso@unina.it

Table of contents:

- Figure S1
- Figure S2
- Figure S3
- Figure S4
- Table S1. TMPRSS2 locus region variants
- Table S2. TMPRSS2 coding variants
- Table S3. TMPRSS2 eQTL lung variants
- Table S4. TMPRSS2 eQTLs
- Table S5. Populations (https://macarthurlab.org/2018/10/17/gnomad-v2-1/)
- Table S6. TMPRSS2 variants from GWAS analysis (DOI: 10.1056/NEJMoa2020283)

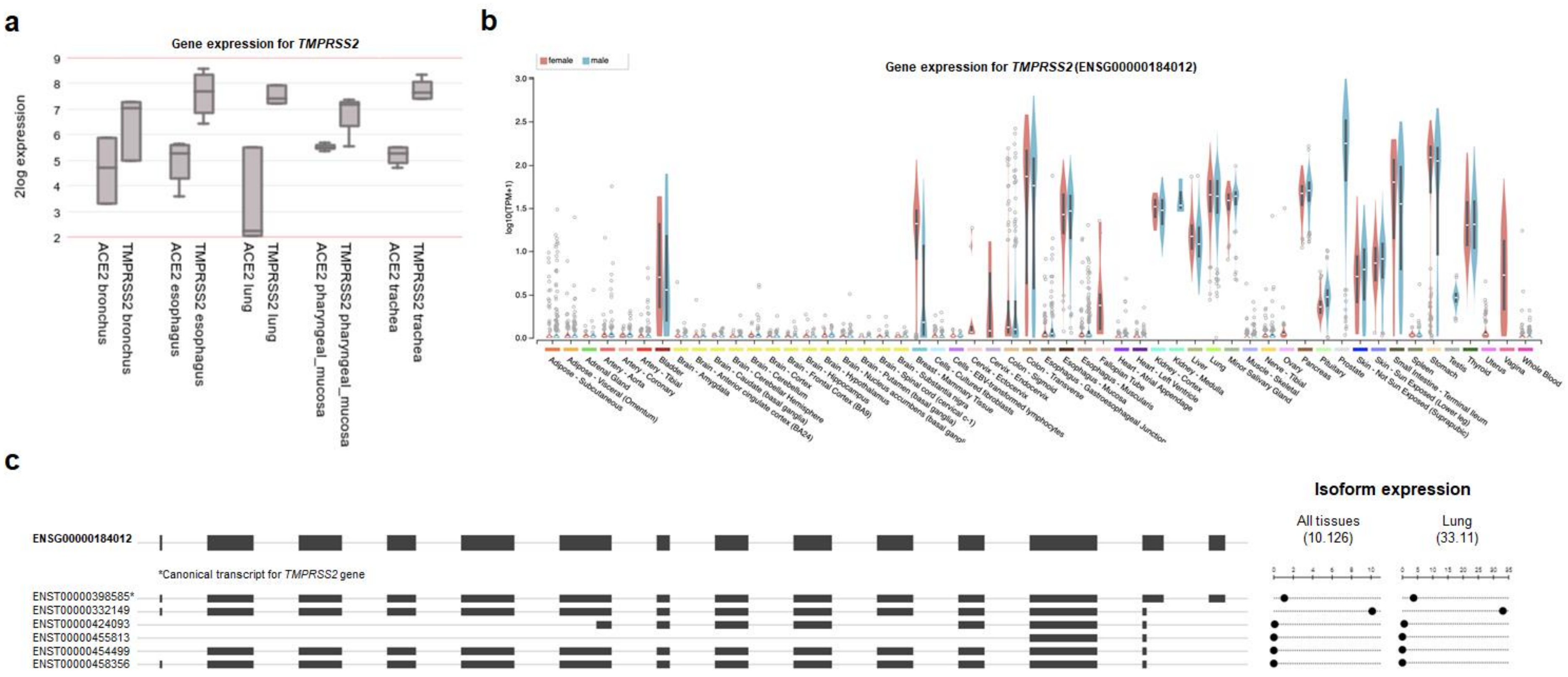

Figure S1. Gene expression analysis on *TMPRSS2* gene.

a) Expression analysis of the *TMPRSS2* and *ACE2* genes by R2, a web-based genomic analysis and visualization tool (R2: Genomics Analysis and Visualization Platform, http://r2.amc.nl). Box plot of each gene expression obtained by the dataset from normal and tumor tissues. Data are shown as 2log expression. **b)** Gene expression profile of *TMPRSS2* gene in different tissues and stratified according to the gender, as obtained from Genotype Tissue Expression (GTEx) database (https://www.gtexportal.org/home). **c)** Schematics of *TMPRSS2* gene and transcripts as obtained from gnomAD database (https://gnomad.broadinstitute.org/).

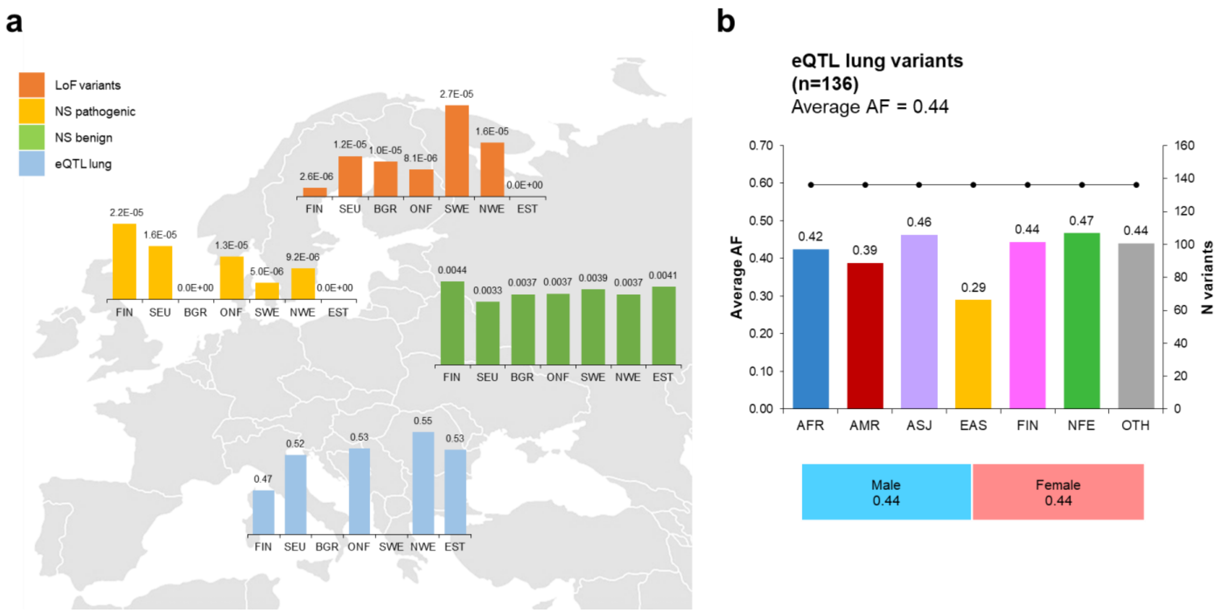

Figure S2. Allele frequency distribution of coding variants and eQTLs-lung of *TMPRSS2* in different populations.

a) The average allele frequency distribution of non-synonymous pathogenic, benign, loss-of-function, and eQTLs-lung (positive NES) of TMPRSS2 in different European populations. The colors of each histogram indicate different types of variants as shown in the color code legend. FIN, Finnish; SEU, Southern European; BGR, Bulgarian; ONF, Other non-Finnish European; SWE, Swedish; NEW, North-Western European; EST, Estonian.

b) The allele frequency distribution of eQTL of *TMPRSS2* in different European populations in different populations. The Y axis of the bar plot shows average allele frequency (AF) in each population. The second right axis displays the number of variants within each population. The bar graph below shows the AF distribution in the overall gnomAD population stratified according to gender. AFR, African/African American; AMR, Latino/Admixed American; ASJ, Ashkenazi Jewish; EAS, East Asian; FIN, Finnish; NFE, Non-Finnish European; SAS, South Asian; OTH, Other (population not assigned).

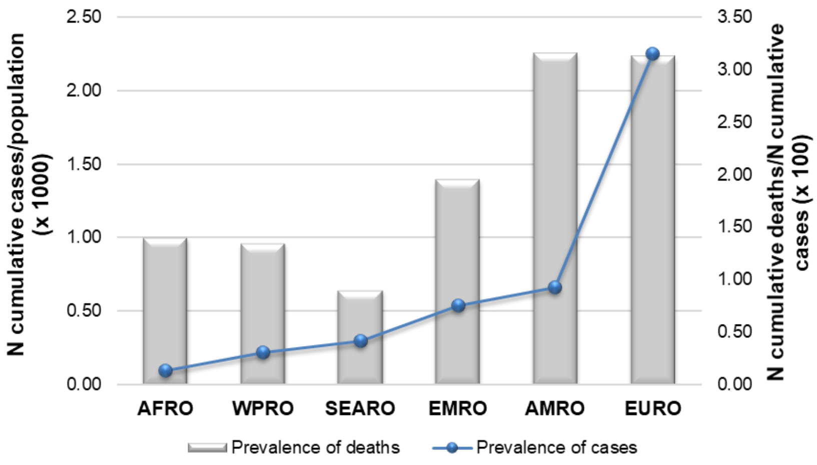

Figure S3. COVID-19 epidemiology by WHO region.

The cumulative number of COVID-19 cases and deaths were derived from the WHO Coronavirus Disease (COVID-19) Dashboard (https://covid19.who.int/; data last update: June 29, 2020). The median prevalence of COVID-19 cases in each WHO region was calculated by the cumulative number of COVID-19 confirmed cases normalized on the population 2020 of each country in the world (https://www.worldometers.info/world-population/population-by-country/). The death rate in each WHO regions has been assessed by the ratio of cumulative number of deaths normalized on the number of cumulative cases.

AFRO, Africa; AMRO, Americas; EMRO, Eastern Mediterranean; EURO, Europe; SEARO, South East Asia; WPRO, Western Pacific.

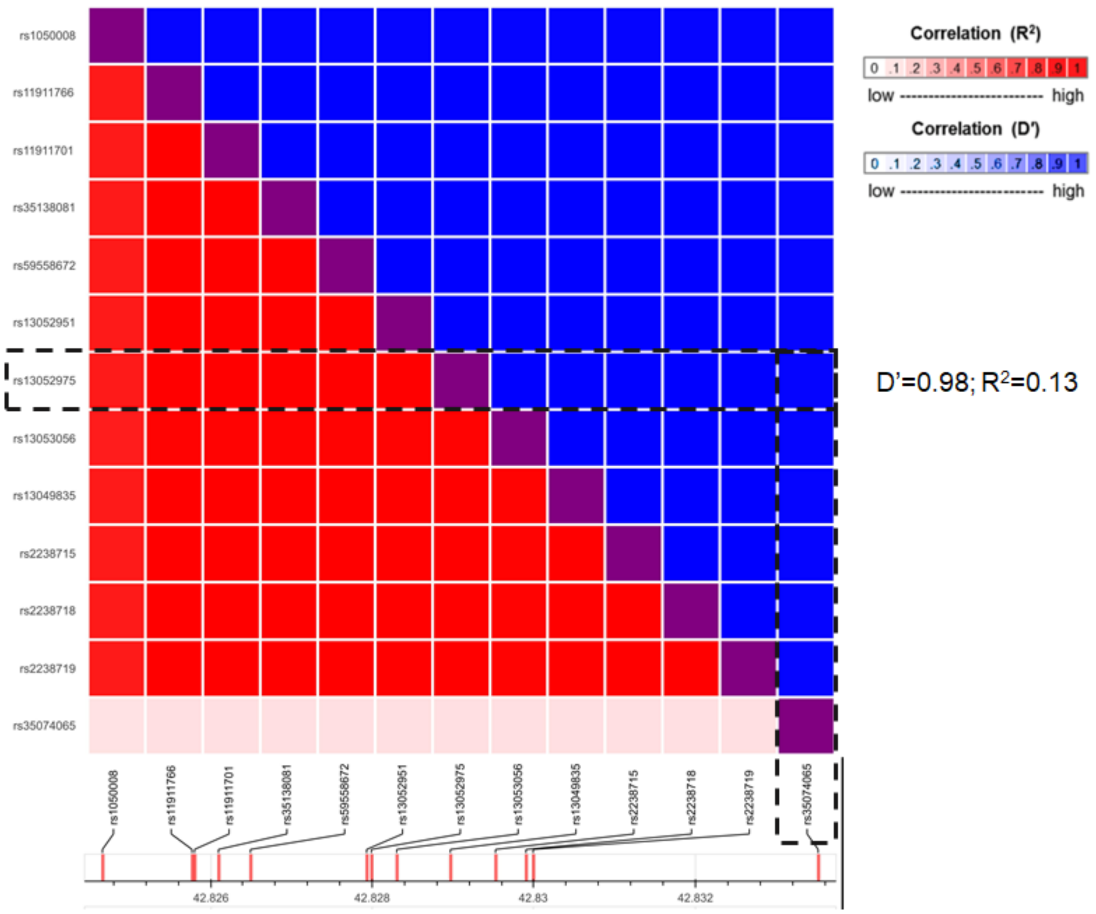

Figure S4. Linkage disequilibrium block at *TMPRSS2* locus of GWAS data

Linkage disequilibrium between the top 13 SNPs, nominally significantly associated with the development of severe COVID-19, and the top significant eQTL for TMPRSS2 in lung tissue. The two top SNPs (rs13052975 and rs35074065) are highlighted in the black boxes. The D' and R2 data are computed with the genetic information from European population by using the web tool LD-link (https://ldlink.nci.nih.gov/?tab=home).
